# Peri‐implantation urinary hormone monitoring distinguishes between types of first‐trimester spontaneous pregnancy loss

**DOI:** 10.1111/ppe.12613

**Published:** 2020-02-13

**Authors:** Lin Foo, Sarah Johnson, Lorrae Marriott, Tom Bourne, Phillip Bennett, Christoph Lees

**Affiliations:** ^1^ Institute for Reproductive and Developmental Biology Imperial College London UK; ^2^ SPD Development Company Ltd Bedford UK

**Keywords:** biochemical pregnancy, hCG, miscarriage, ovulation

## Abstract

**Background:**

Lutenising hormone (LH) and human chorionic gonadotropin (hCG) hormone are useful biochemical markers to indicate ovulation and embryonic implantation, respectively. We explored “point‐of‐care” LH and hCG testing using a digital home‐testing device in a cohort trying to conceive.

**Objective:**

To determine conception and spontaneous pregnancy loss rates, and to assess whether trends in LH‐hCG interval which are known to be associated with pregnancy viability could be identified with point‐of‐care testing.

**Methods:**

We recruited healthy women aged 18‐44 planning a pregnancy. Participants used a home monitor to track LH and hCG levels for 12 menstrual cycles or until pregnancy was conceived. Pregnancy outcomes (viable, clinical miscarriage, or biochemical pregnancy loss) were recorded. Monitor data were analysed by a statistician blinded to pregnancy outcome.

**Results:**

From 387 recruits, there were 290 pregnancies with known outcomes within study timeline. Adequate monitor data for analysis were available for 150 conceptive cycles. Overall spontaneous first‐trimester pregnancy loss rate was 30% with clinically recognised miscarriage rate of 17%. The difference to LH‐hCG interval median had wider spread for biochemical losses (0.5‐8.5 days) compared with clinical miscarriage (0‐5 days) and viable pregnancies (0‐6 days). Fixed effect hCG profile change distinguished between pregnancy outcomes from as early as day‐2 post‐hCG rise from baseline.

**Conclusions:**

The risk of first‐trimester spontaneous pregnancy loss in our prospective cohort is comparable to studies utilising daily urinary hCG collection and laboratory assays. A wider LH‐hCG interval range is associated with biochemical pregnancy loss and may relate to late or early implantation. Although early hCG changes discriminate between pregnancies that will miscarry from viable pregnancies, this point‐of‐care testing model is not sufficiently developed to be predictive.

**Synopsis**

1 **Study question**

Can a home digital device detect biochemical changes that are associated with first‐trimester miscarriage?

2 **What is already known**

The incidence of first‐trimester spontaneous pregnancy loss is frequently quoted at 25%, and this is thought to be an under‐estimation due to very early gestational losses that may be missed. Using methodologies that are time and resource‐heavy, delayed timing of embryonic implantation has been shown to be associated with first‐trimester miscarriage.

3 **What this study adds**

First‐trimester spontaneous pregnancy loss in this prospective cohort was 30%. A home end‐user device is feasible to study hormonal trends associated with pregnancy loss, including early or late implantation. Significantly, the device detected early changes in hCG that may be able to distinguish between types of pregnancy loss from day 1 post rise from baseline.

## 1 INTRODUCTION

Observing rates of conception and pregnancy loss are important in understanding reproductive health. Low clinical pregnancy rates may be due to decreased conception rates, increased spontaneous pregnancy loss, or both. Very early pregnancy loss, occurring before 6 gestational weeks of pregnancy, is often not clinically apparent and is often mistaken for heavier menstrual loss, which can confound rates of fecundity or miscarriage. Using pre‐ and peri‐implantation biochemical markers to assess pregnancy viability may be particularly valuable in the first few weeks of pregnancy, when ultrasound imaging is frequently not informative.^1^, ^2^

Prior to the conception, an acute rise of luteinising hormone (LH) produced by the anterior pituitary gland triggers ovulation within 22‐24 hours.^3^ Therefore, day of conception (ie fertilisation of the embryo, as the unfertilised ovum has a lifespan of less than 1 day) can be assumed to be on the day following the LH surge. Human chorionic gonadotropin (hCG), a hormone produced by the trophoblast cells of the developing embryo, is universally used as a pregnancy biomarker in laboratory and commercial tests.^4^ In viable pregnancies, levels of hCG rise consistently with respect to time from conception through early pregnancy.^5^, ^6^ By day 35 following ovulation, mean urinary concentrations vary between 65 000 pmol/L and 2 700 000 pmol/L.^7^ A slower rise in log hCG is associated with early pregnancy loss.^8^, ^9^

The first detection of serum hCG is often taken to reflect implantation day, and significantly, a prolonged interval between ovulation and implantation is associated with spontaneous early pregnancy loss.^10^, ^11^ Therefore, both hormone trajectories provide a potential biochemical basis for determination of pregnancy viability.

To accurately define very early peri‐conception biochemical changes requires women to be prospectively studied from the beginning of non‐contraception cycles. This is methodologically complex as women must be recruited prospectively, approximately 25% of pregnancies are unplanned, and women recruited pre‐conception often have a low follow‐up rate during pregnancy due to the demands placed on them by the timescale involved in both conceiving and pregnancy.^12^, ^13^ Hence, few prospective studies have observed both conception and pregnancy loss rates per cycle.^2^, ^14^, ^15^ Most use retrospective analysis of biochemical tests, or study conceptions arising from assisted reproductive technologies (ART). Prospective monitoring of conception cycles and pregnancy loss in spontaneous pregnancy has been studied by the collection of daily urinary samples and refrigerating or freezing the samples before they are sent for analysis.^2^, ^11^ This methodology is often time‐ and resource‐consuming and is unlikely to be practical for general monitoring. Recently, studies have utilised ovulation monitors to monitor conception and pregnancy loss rates at home, rather than through laboratory testing.^16^

In this study, we investigated conception and spontaneous first‐trimester pregnancy loss rates within a prospective healthy cohort, using a digital home‐testing fertility monitor that allowed “point‐of‐care” testing. Our study protocol had an additional advantage of ascertainment and analysis of semi‐quantitative hormonal concentration data from the monitors. This facilitated more accurate detection of the events and timing of ovulation, embryonic implantation, and spontaneous pregnancy loss.

Study objectives were to determine conception and pregnancy loss rates and to assess whether known trends in LH and hCG changes, as biochemical markers of ovulation and implantation which have been reported in relation to pregnancy viability, can be replicated using digital home monitors. A post hoc analysis of hCG changes in relation to pregnancy outcome was also performed.

## 2 METHODS

We prospectively recruited women aged 18‐44 who planned to conceive a pregnancy within 12 months of study entry. This was part of a larger study investigating cardiovascular changes in pregnancy based at Imperial College London (CONCEIVE Study).^17^, ^18^

### 2.1 Cohort selection

All women were healthy non‐smokers with body mass index <35 and no cardiovascular co‐morbidities including hypertension, diabetes, thrombophilia, or renal disease. We excluded women who had irregular cycles (>42 days cycle length), recurrent miscarriage (>3 consecutive miscarriages), or those who were pursuing conception involving medical assistance. Participants had stopped contraceptive methods at least one month prior to study entry and had not been using a long‐acting contraception.

Participants were recruited via the use of local and social media platforms as well as poster distributions to local general practice surgeries, community centres, hospitals, and the university at which the study was based.

All participants attended a first study visit at which time written consent was obtained, and height and weight measured from which body mass index (BMI) was derived. Participants completed a detailed health questionnaire providing details of obstetric history including all outcomes of previous conceptions and duration of trying for a pregnancy. All were screened for pregnancy using a urinary HCG pregnancy test stick, and those with a positive result were excluded from study entry.

Participants were then provided with a digital home ovulation and pregnancy testing monitor (Clearblue Advanced Fertility Monitor, SPD Development Company Ltd, Bedford, UK) to use for a maximum of 12 months as a research monitor to collect data, and also to enable volunteers to time intercourse to the fertile window. The monitor tracked and stored levels of urinary oestrogen and LH from fertility test sticks and enabled participants to identify their most fertile days to optimise chances of conceiving a pregnancy.^19^ Separate test sticks were used to measure hCG levels to detect pregnancy. Participants were instructed to follow the testing protocol described below. This protocol was specifically designed for the study to ensure data on both LH surge and first rise of hCG from baseline were collected.

### 2.2 Fertility monitor testing protocol

Participants input the date of the first day of their menstrual cycle (LMP) into the monitor within 4 days of their cycle commencing, in order to “set up” monitor calibration. They then started daily testing with fertility sticks on their first morning urine, from cycle day 6.

The output from the monitor consisted of three readings. A “low” reading indicated that oestrogen and LH levels were still at baseline, and a “high” reading indicated that the monitor had detected a rise oestrogen, which occurs approximately 72 hours before ovulation. A “peak” reading indicated an LH surge which has been demonstrated to predict ovulation within 22 hours (±1 day),^20^ and the “peak” reading is displayed to the user for 2 consecutive days. Participants were advised to try for a pregnancy when they had “high” and “peak” readings to optimise the chances of conception. On the first day, a “peak” reading was obtained, and participants stopped ovulation testing and commenced pregnancy testing using the same monitor (but with a pregnancy testing stick) from 7 days following a “peak” until they obtained positive pregnancy test results over at least 3 consecutive days or their next menstrual cycle started.

Once a participant had obtained consecutive positive pregnancy test results, she was instructed to contact the research team, and a follow‐up appointment at 6 weeks of gestation was arranged, calculated from first day of LMP.

### 2.3 Follow‐up visits

At the follow‐up visit at 6 weeks from LMP, participants underwent a trans‐vaginal ultrasound scan to assess location and viability of the pregnancy, and scan outcomes were classified to following outcomes: viable intrauterine pregnancy (IUP), pregnancy of unknown viability (PUV) where a visible intrauterine gestation sac was visualised but with no other indicators of pregnancy viability (embryonic cardiac activity) or pregnancy of unknown location (PUL), where no ultrasound evidence of pregnancy was visualised. PUV and PUL were followed up in accordance with local early pregnancy guidelines until viability of the pregnancy was confirmed. A ultrasound diagnosis of non‐viable pregnancies was made in accordance with the Royal College of Obstetricians & Gynaecologists Guidelines (2011) on the basis of a mean gestation sac diameter ≥25 mm (with no obvious yolk sac), or with an embryo with no visible heartbeat and a crown‐rump length of ≥7 mm.^21^

Non‐viable pregnancies were then further classified as per European Society of Human Reproduction and Embryology (ESHRE) guidance.^22^

At the same visit, the fertility monitor was returned to the research team and data relating to ovulation and pregnancy testing were extracted and analysed at SPD Development Company Ltd. The level of intensity of the LH and hCG test lines on the lateral flow test sticks, which is not available to users, was downloaded along with the timing of tests and LMP data. The test line intensity is directly related to the concentration of hormone in the sample (up until line saturation) enabling semi‐quantitative examination of hormone levels from the research monitors. The hCG assay measured intact hCG, and line intensity has a linear association with hCG concentration in the first week that hCG becomes detectable. The limit of detection is approximately 2 mIU/mL, but a positive pregnancy test result is only provided to the user at 50 mIU/ml. This means that several days of hCG signal data are collected prior to a volunteers 3 consecutive positive HPT results, providing sufficient data for longitudinal analysis. Laboratory investigators at SPD were blinded to all clinical information during data extraction and analyses.

### 2.4 Clinical outcome definitions

Time to clinical pregnancy was defined as the number of menstrual cycles from the date of study entry to clinical pregnancy.

Pregnancy loss was defined as per the ESHRE consensus statement^22^:

- Biochemical pregnancy loss: Spontaneous pregnancy demise based on decreasing serum or urinary b‐HCG levels, without an ultrasound evaluation.

Participants who had a positive pregnancy test but lost the pregnancy (in all cases as they had bleeding followed by a negative hCG pregnancy test) before the study follow‐up visit at 6 weeks of gestation were classified as a biochemical pregnancy loss.

- Clinical Miscarriage: Intrauterine pregnancy demise confirmed by ultrasound or histology

### 2.5 Statistical analyses

A panel consisting of three clinicians, a SPD scientific advisor, and a statistician reviewed downloaded data for each cycle. By panel consensus, the day of the LH surge was assigned to monitor “peak” day and hCG signal data were also reviewed graphically to determine the first rise from baseline.

Additionally, a post hoc analysis was undertaken. For each outcome status, a random effects longitudinal model was fitted to the hCG signal data, generating a set of intercepts and slopes, one for each pregnancy. Due to the nature of the test assay, which will saturate and plateau, a quadratic (squared) term was included in the model to allow for this curvature. To examine differences between the outcome status models, a comparison of the random effects estimates for the intercept, slope, and quadratic terms was assessed by ANOVA and the estimates of differences and 95% confidence intervals were calculated. The time point in which a difference in hCG signal between the outcome groups was assessed by multiple ANOVAs of the predicted hCG for each time point with estimates of the differences and 95% confidence intervals.

Data were analysed using SAS 9.3 software.

### 2.6 Ethics approval

The study received ethical approval from the East of Scotland Research Ethics Service (REC reference: 14/ES/1046) and local Research and Development Authority (Imperial Joint Research Compliance Office, London; Ref: 14HH2169).

## 3 RESULTS

We recruited 387 women over 22 months, of whom 28 women withdrew for reasons including having changed their mind about conceiving, stress of the testing protocol, and moving out of the study locality. Six were excluded as they were subsequently found to have been pregnant at study entry. Of the remaining 353 women, there were 293 conceptions in a total of 254 women giving a conception rate of 72%. During the study, several women had more than one pregnancy; for example, they conceived a pregnancy that ended in miscarriage and then re‐entered the study and conceived another pregnancy. There were 221 women who had only one pregnancy, 30 women who had 2 pregnancies, and 3 women who had 3 pregnancies. The demographics for all study participants (regardless of whether conception was achieved) are summarised in Table 1.

**Table 1:** Demographics for whole study cohort, and for the 150 participants in which adequate monitor data for conceptive cycles were available for analyses, categorised by first‐trimester pregnancy outcomes

Characteristics	Total recruited	Subgroup with monitor data, split by pregnancy outcome		
		Viable mean (SD)	Biochemical pregnancy loss mean (SD)	Clinical miscarriage mean (SD)
Participants (number)	387	97	27	24
Conceptions (number)	293	97	28*	25*
Maternal age (years)	34.0 (3.8)	34.1 (4.0)	36.3 (3.9)	36.7 (4.3)
Maternal BMI (kg/m^2^)	23.9 (15.3)	23.4 (17.7)	23.3 (12.4)	22.9 (16.6)
Nulliparity (%)	60	53	46	60
Ethnicity (n)				
White Caucasian	266	74	22	19
South Asian	61	13	4	3
Afro‐Caribbean	43	5	0	2
Other	17	5	1	0

* Denotes 1 participant had 2 conceptions within the same pregnancy outcome category.

Prior to entry into the study, participants had been trying for an average of 2.59 months to conceive a pregnancy (SD 2.99). After entry into the study and with the use of the fertility monitors, the interval to conceiving a pregnancy was 3.09 months (SD 3.73). The overall total average time trying to conceive including the time before and after study entry was 5.23 months (SD 4.54).

Monitor data extracted from conceptions were assessed using a stringent protocol to identify cycles that had sufficient data to define cycle days for both ovulation and implantation. As a minimum, data (ie testing) were available for a day before and following an identifiable LH surge, and at least 2 identifiable days of a hCG baseline. As some participants were non‐compliant with the testing protocol, there was adequate monitor data for 150 conceptions. The subgroup with monitor data was very similar to the wider population. The participant demographics for conceptions in which monitor data were utilised are presented in Table 1, split by first‐trimester pregnancy outcomes. Maternal age was higher in the clinical miscarriage and biochemical pregnancy loss groups compared with viable pregnancies group, but no other demographic variables were appreciably different. Of note, 2 participants had two index conceptive cycles that had sufficient data that were included for analyses. Both remained within the same outcome group as their pregnancy outcomes were the same (one participant had 2 biochemical pregnancy losses, and one had 2 clinical miscarriages).

### 3.1 Pregnancy loss within the cohort

For all conceptions within the study, with regard to end pregnancy outcomes, three pregnancies were lost to follow‐up, four pregnancies were terminated for aneuploidy, and two were ectopic pregnancies. Of the pregnancies in which outcomes were known (290 pregnancies), there were 87 first‐trimester spontaneous pregnancy losses (30%) and 2 second‐trimester spontaneous pregnancy losses. Spontaneous first‐trimester loss was further divided into biochemical pregnancy loss and clinical miscarriage. Thirty‐eight conceptions ended in biochemical pregnancy loss, which accounted for 13% of pregnancies with known outcomes that may otherwise have been clinically unrecognised. Accordingly, the first‐trimester clinical miscarriage rate was 17%. The average gestation for first‐trimester loss was 5 weeks + 1‐day of gestation. If only clinically recognised pregnancy loss was considered, the average gestation for loss was 7 weeks + 6 days of gestation.

### 3.2 LH‐hCG interval

There were sufficient biochemical data available for 150 of 290 conceptions with known pregnancy outcomes. For these 150 conceptions, the time between LH surge and hCG rise (LH‐hCG interval) between pregnancies that were viable beyond the first trimester, and those that miscarried were compared. Overall, there were no significant differences between the intervals of the outcome groups with viable pregnancy mean: 9.27 days (min‐max 7‐15), clinical miscarriage mean: 9.46 days (min‐max 7‐14), and biochemical pregnancy loss mean: 8.72 days (min‐max 1‐13). Median intervals were 9 days in both the viable and clinical miscarriage groups and 9.5 days in the biochemical pregnancy group. However, the range of intervals between the groups, taken as the difference to the median, shows a wider spread of intervals in the biochemical losses compared with the clinical miscarriage and viable pregnancies. The total range of distance from the median (minimum to maximum) was 0‐6 days (median 1 day) for viable pregnancies, 0.5‐8.5 days (median 2 days) for biochemical pregnancy losses, and 0‐5 (median 1 day) for clinical miscarriages. Absolute differences were examined without consideration of direction in order to assess overall extent of variability. ANOVA comparisons are shown in Table 2.

**Table 2:** ANOVA comparison of median days from median LH‐hCG interval based on first‐trimester pregnancy outcome

Outcome comparison		Difference (95% confidence interval)
Viable	Clinical miscarriage	−0.01 (−0.75, 0.72)
Viable	Biochemical pregnancy loss	−1.32 (−2.24, −0.40)
Clinical miscarriage	Biochemical pregnancy loss	−1.30 (−2.37, −0.24)

### 3.3 hCG profiles

**Figure 1:**
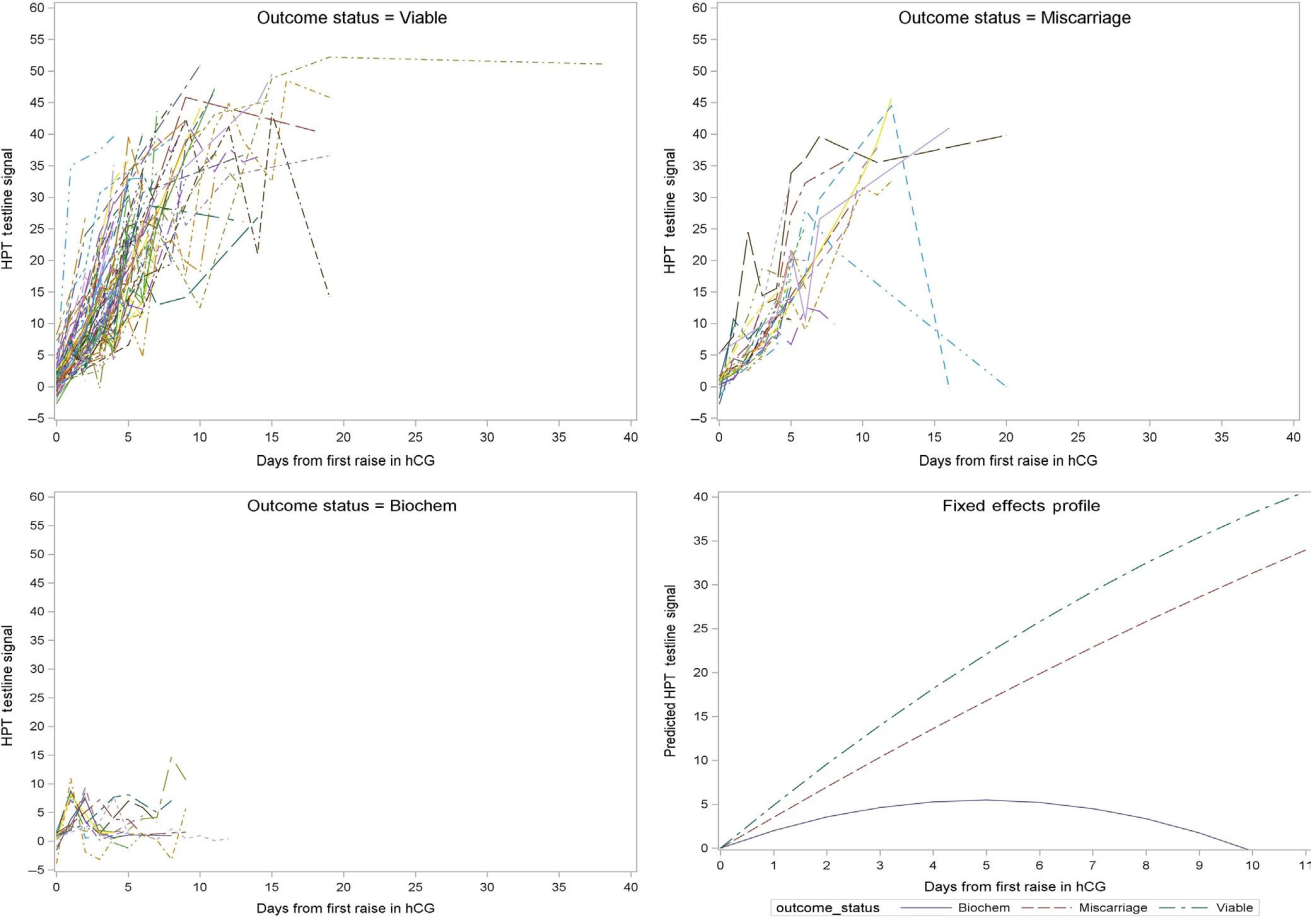
Digital monitor HPT (home pregnancy test) test line signal data for biochemical, miscarriage, and viable pregnancies and fixed effect profile models for each group. HPT test line signal data equates to the amount of hCG present in the urine sample tested

The daily hCG test signal levels for each pregnancy are shown in Figure 1, for each cohort. These were used to create longitudinal models for each pregnancy cycle, with respect to the first emergence of hCG. The random effects estimates for the intercept, slope, and quadratic term for each cohort's model were compared to characterise the functional form of the hCG rise and determine whether there were fundamental differences between groups (Table 3). Biochemical pregnancy losses had a smaller slope and larger quadratic terms, indicating a slower rise in hCG, followed by a quick decline. Although the curves appeared markedly different, these differences were not statistically significant.

**Table 3:** Random effects estimates for the intercept, slope, and quadratic terms assessed by ANOVA for specified clinical outcomes of viable pregnancy, clinical miscarriage, and biochemical pregnancy loss

Outcome comparison		Difference (95% confidence interval)		
		Intercept	Linear	Quadratic
Viable pregnancy	Clinical miscarriage	0.01 (−1.01, 1.02)	−0.03 (−0.92, 0.86)	0.01 (−0.07, 0.08)
Viable pregnancy	Biochemical pregnancy loss	−0.31 (−1.57, 0.95)	0.91 (−0.20, 2.02)	−0.09 (−0.18, 0.01)
Clinical miscarriage	Biochemical pregnancy loss	−0.32 (−1.78, 1.14)	0.94 (−0.35, 2.23)	−0.09 (−0.20, 0.01)

Estimates of differences and 95% confidence intervals are shown for each pairwise comparison.

Table 4 shows the fixed effects hCG signal profiles for each cohort calculated from the model. Day 0 is the day on which the hCG signal first exceeds baseline, and on this day, there is no difference between the groups. However, from day 1 following the hCG rise, there is a significant difference between the viable and biochemical pregnancy loss cohort, and from day 2, there is a difference between all three pregnancy outcomes which persists through to the last day examined in this study.

**Table 4:** Differences in hCG signal intensity by day of pregnancy for viable pregnancy, clinical miscarriage, and biochemical pregnancy loss

Day relative to first rise in hCG	Outcome comparison		Difference (95% confidence interval)
0	Viable	Clinical miscarriage	0.006 (−1.006, 1.017)
	Viable	Biochemical pregnancy loss	−0.314 (−1.574, 0.946)
	Clinical miscarriage	Biochemical pregnancy loss	−0.320 (−1.784, 1.144)
1	Viable	Clinical miscarriage	1.444 (−0.222, 3.120)
	Viable	Biochemical pregnancy loss	3.597 (1.618, 5.577)
	Clinical miscarriage	Biochemical pregnancy loss	2.154 (−0.166, 4.475)
2	Viable	Clinical miscarriage	2.329 (0.202, 4.457)
	Viable	Biochemical pregnancy loss	6.952 (4.345, 9.558)
	Clinical miscarriage	Biochemical pregnancy loss	4.622 (1.559, 7.685)
3	Viable	Clinical miscarriage	3.163 (0.401, 5.924)
	Viable	Biochemical pregnancy loss	10.783 (7.399, 14.168)
	Clinical miscarriage	Biochemical pregnancy loss	7.620 (3.707, 11.534)
4	Viable	Clinical miscarriage	3.353 (0.197, 6.508)
	Viable	Biochemical pregnancy loss	13.972 (10.257, 17.687)
	Clinical miscarriage	Biochemical pregnancy loss	10.619 (6.235, 15.003)
5	Viable	Clinical miscarriage	2.733 (−0.219, 5.685)
	Viable	Biochemical pregnancy loss	16.688 (13.184, 20.196)
	Clinical miscarriage	Biochemical pregnancy loss	13.954 (9.876, 18.033)

## 4 COMMENT

### 4.1 Principal findings

First‐trimester spontaneous pregnancy loss rate including biochemical pregnancy loss is 30%, with a clinical miscarriage rate of 18%. Using a commercially available monitor, we were able to identify early biochemical losses that may otherwise have been clinically unrecognised.

While a long LH‐hCG interval has previously been reported to be associated with miscarriage, we report that first‐trimester spontaneous pregnancy loss was associated with both short and long intervals, with a wider spread of intervals from the median.

Further, we observed from that that profiles change in fixed effect hCG signals over time could distinguish between viable, biochemical pregnancy loss, and clinical miscarriages.

### 4.2 Strengths of the study

A strength of our study is prospective methodology, and participant biometrics were clinically collected, rather than self‐reported, so there is greater accuracy of data. Additionally, the entry criteria to study were stringent; hence, only women who were healthy and not known to have primary or secondary sub‐fertility were included. Further, semi‐quantitative measurements of hCG and LH were obtained in real time by participants as the sample was obtained, as opposed to delayed testing of a sample which has been refrigerated/frozen and then transported to a laboratory.

### 4.3 Limitations of the data

A limitation of our study is the proportion of missing monitor data due to inconsistent testing in some of our participants, which could have altered the “fit” of the model, although the extent of this is unknown. The missing data may reflect the variability in end‐user (and perhaps consumer) testing with the digital fertility monitors. Further, as we excluded women with recurrent miscarriages, as well as those who suffered from sub‐fertility, thereby this could affect the generalisability of results reported here and hence their application to a wider population.

### 4.4 Interpretation

First‐trimester spontaneous pregnancy loss rate reported in our study is significantly higher than the range frequently quoted during standard patient counselling of 20%.^23^, ^24^, ^25^ Our data suggest that this may be an underestimate and concur with figures reported by Wilcox^2^ who had used daily urinary collection and a hCG immune‐radiometric assay that detected hCG levels as low as 0.01 ng per millilitre. These figures are also comparable with other pre‐conception studies that utilised daily urinary assays or home monitoring of hCG via a commercial monitor, overall indicating that true pregnancy loss rate is approximately one‐third of all conceptions.^16^

Analysis of fixed effect hCG profile has not previously been reported in relation to pregnancy outcome. Our findings suggest it is theoretically feasible to develop a model to assess pregnancy viability as early as day 1 following embryonic implantation, as viable and biochemical pregnancy loss signals differed significantly. From day 2, all three outcomes viable, biochemical pregnancy loss, and clinical miscarriage can be distinguished from one another, though the confidence intervals are wide. These data are insufficient to develop a robust predictive model, serving rather as a proof of principle.

Results of LH‐hCG intervals in associations with first‐trimester spontaneous pregnancy loss suggest that there is an optimal time frame for embryonic implantation, and a greater distance in days from this window (shorter or longer) is associated with pregnancy loss.

Our study design demonstrates that a low‐resource methodology using a commercially available point‐of‐care device could replicate findings that have previously required expensive and time‐consuming data collection protocols.

Further, the findings suggest that in principle at least women might have access to immediate information regarding first‐trimester spontaneous pregnancy loss, though the model that we propose is currently not suitable for translation to a population setting. Whether such information would be beneficial in understanding their reproductive health and facilitate referral to specialist services such as recurrent miscarriage clinics if all miscarriages were identified remains speculative.

The consequences of early pregnancy loss being overlooked may not be entirely disadvantageous. Identifying pregnancy earlier runs the risk of women undergoing earlier ultrasound scans leading to more being classified as having a pregnancy of unknown location with the associated repeated blood tests and scans for a condition for which there is no treatment. Furthermore, it is likely that more couples will suffer psychological morbidity associated with early pregnancy loss.^26^ Hence, more knowledge for women in this context may have undesirable sequelae.

## 5 CONCLUSIONS

In conclusion, we report we have established the overall risk of miscarriage including biochemical pregnancies in a prospective cohort. Furthermore, we have demonstrated that distance from the median LH‐hCG interval is associated with first‐trimester spontaneous pregnancy loss and that changes in hCG over time from as early as day 1 post rise from baseline may be able to discriminate between pregnancies destined to miscarry and those that are viable, with biggest differences observed for biochemical pregnancy loss in comparison with viable pregnancies, information which has the potential to be a useful diagnostic test for pregnancy viability.

## CONFLICT OF INTEREST

 SJ and LM are employees of SPD Development Company Ltd., a fully owned subsidiary of SPD Swiss Precision Diagnostics GmbH; the manufacturer of the Clearblue fertility monitor used in this study.
